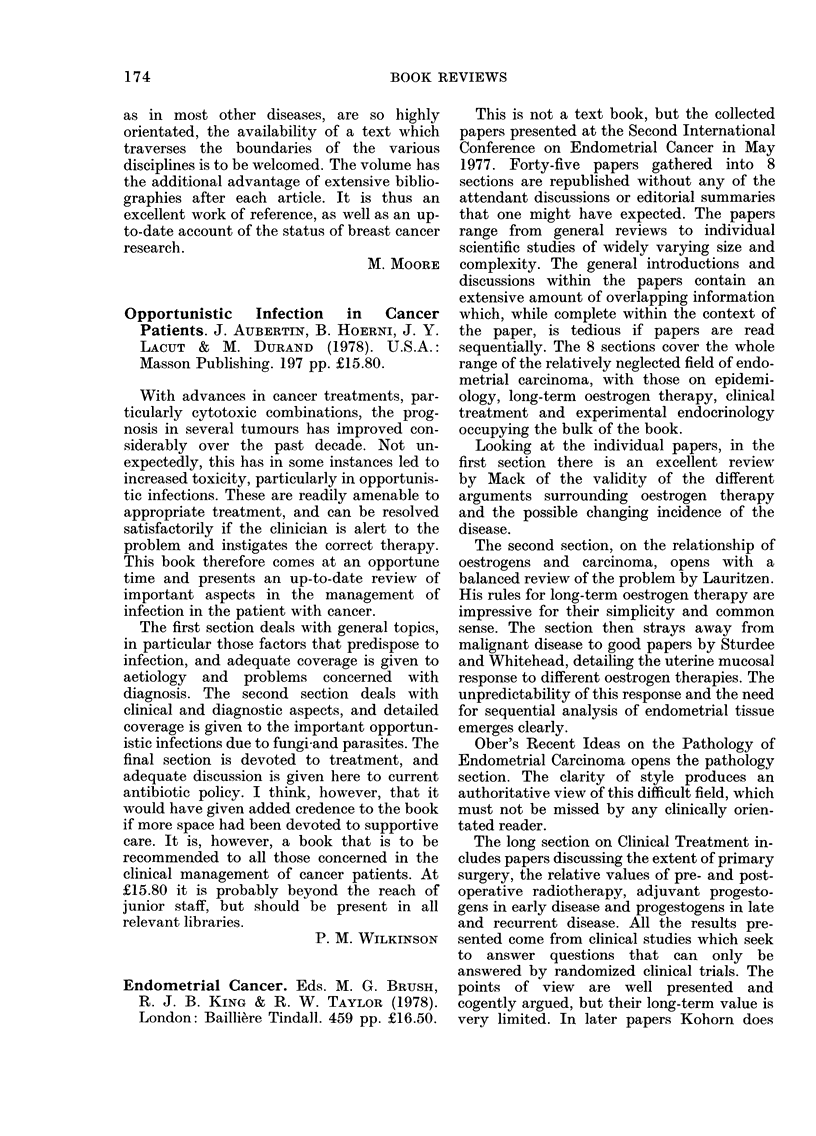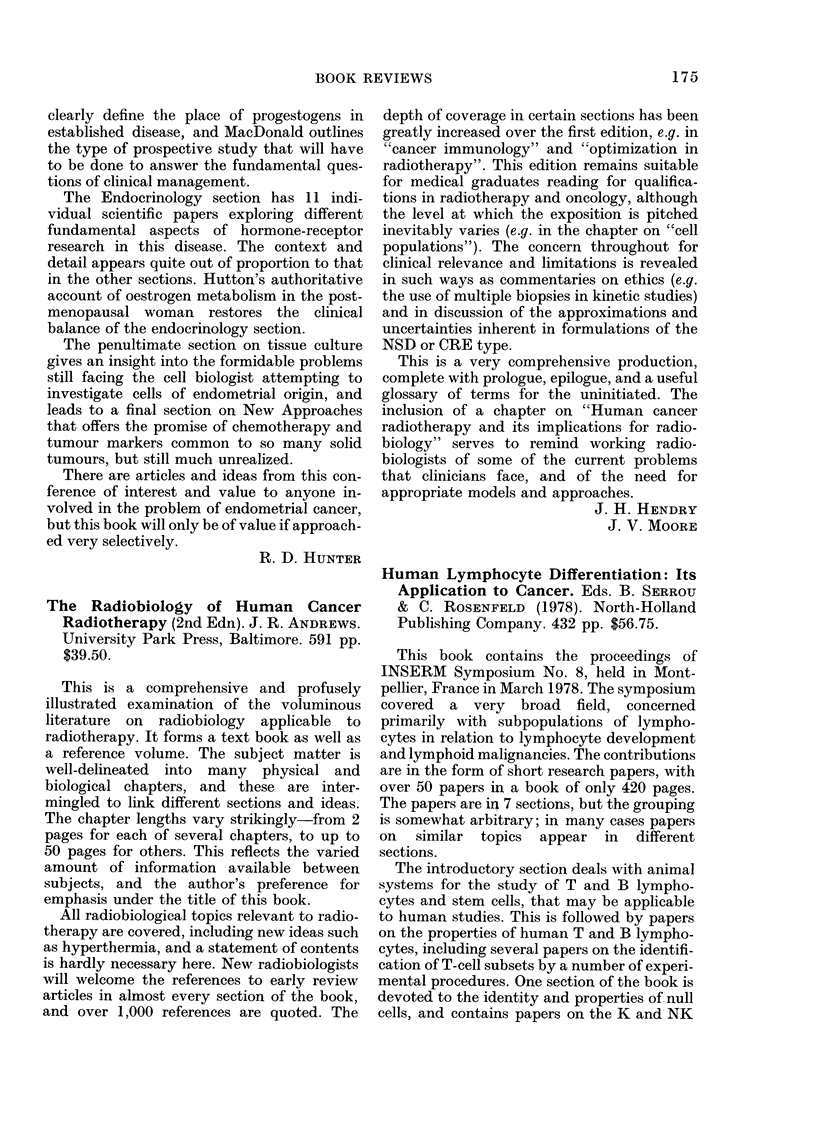# Endometrial Cancer

**Published:** 1979-07

**Authors:** R. D. Hunter


**Endometrial Cancer.** Eds. M. G. BRUSH, R. J. B. KING & R. W. TAYLOR (1978). London: Baillière Tindall. 459 pp. £16.50.

This is not a text book, but the collected papers presented at the Second International Conference on Endometrial Cancer in May 1977. Forty-five papers gathered into 8 sections are republished without any of the attendant discussions or editorial summaries that one might have expected. The papers range from general reviews to individual scientific studies of widely varying size and complexity. The general introductions and discussions within the papers contain an extensive amount of overlapping information which, while complete within the context of the paper, is tedious if papers are read sequentially. The 8 sections cover the whole range of the relatively neglected field of endometrial carcinoma, with those on epidemiology, long-term oestrogen therapy, clinical treatment and experimental endocrinology occupying the bulk of the book.

Looking at the individual papers, in the first section there is an excellent review by Mack of the validity of the different arguments surrounding oestrogen therapy and the possible changing incidence of the disease.

The second section, on the relationship of oestrogens and carcinoma, opens with a balanced review of the problem by Lauritzen. His rules for long-term oestrogen therapy are impressive for their simplicity and common sense. The section then strays away from malignant disease to good papers by Sturdee and Whitehead, detailing the uterine mucosal response to different oestrogen therapies. The unpredictability of this response and the need for sequential analysis of endometrial tissue emerges clearly.

Ober's Recent Ideas on the Pathology of Endometrial Carcinoma opens the pathology section. The clarity of style produces an authoritative view of this difficult field, which must not be missed by any clinically orientated reader.

The long section on Clinical Treatment includes papers discussing the extent of primary surgery, the relative values of pre- and post-operative radiotherapy, adjuvant progestogens in early disease and progestogens in late and recurrent disease. All the results presented come from clinical studies which seek to answer questions that can only be answered by randomized clinical trials. The points of view are well presented and cogently argued, but their long-term value is very limited. In later papers Kohorn does clearly define the place of progestogens in established disease, and MacDonald outlines the type of prospective study that will have to be done to answer the fundamental questions of clinical management.

The Endocrinology section has 11 individual scientific papers exploring different fundamental aspects of hormone-receptor research in this disease. The context and detail appears quite out of proportion to that in the other sections. Hutton's authoritative account of oestrogen metabolism in the post-menopausal woman restores the clinical balance of the endocrinology section.

The penultimate section on tissue culture gives an insight into the formidable problems still facing the cell biologist attempting to investigate cells of endometrial origin, and leads to a final section on New Approaches that offers the promise of chemotherapy and tumour markers common to so many solid tumours, but still much unrealized.

There are articles and ideas from this conference of interest and value to anyone involved in the problem of endometrial cancer, but this book will only be of value if approached very selectively.

R. D. HUNTER